# Supplementary material for: Adaptations by the coral Acropora tenuis confer resilience to future thermal stress
Source: Commun Biol. 2022 Dec 14;5:1371. doi: 10.1038/s42003-022-04309-5 (PMC9751277; doi:10.1038/s42003-022-04309-5)
Supplement: Supplementary file 2 — Supplementary Figure 1, Table 1 to 6 [file 42003_2022_4309_MOESM2_ESM.pdf]

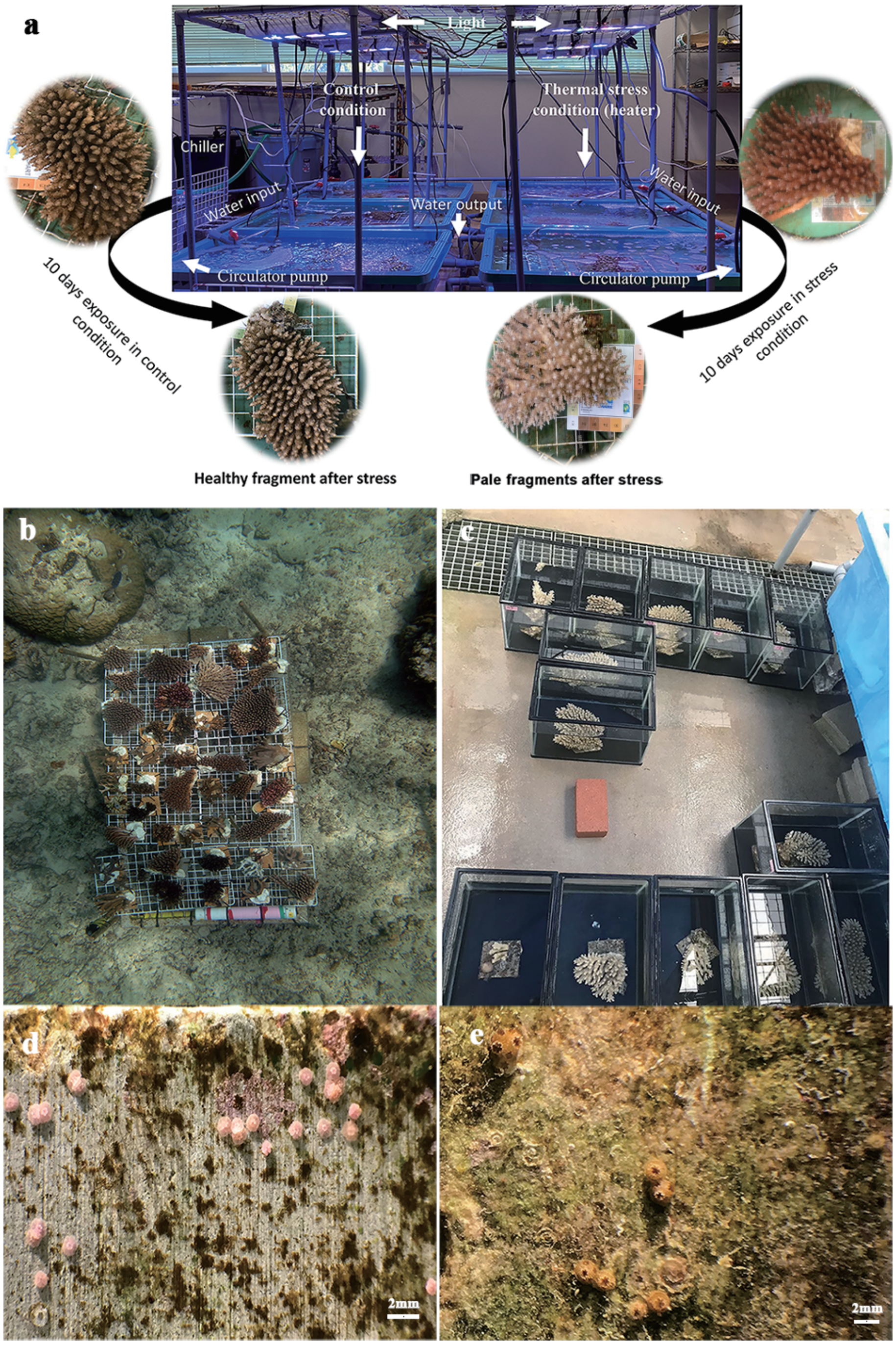

Supplementary Figure 1. a Adult experiment setup and coral fragments in different treatments before and after of experiment. **b** Coral fragments from different treatments (CS and SF) on the frame in the sea after the thermal stress experiment. **c** Coral fragments from different treatments were kept in an individual aquarium for spawning. **d** Settled larvae. **e** Two-month-old recruits.

Supplementary Table 1. Kaplan-Meier log-rank tests for comparisons of larval survival between stressed fragments (SF) and control fragments.

**Contrast**	***p*-value**
**CC-CS**	1.80E-05*
**CC-SC**	0.5719
**CC-SS**	0.00044*
**CS-SC**	0.0001*
**CS-SS**	0.37768
**SC-SS**	0.00239*

Supplementary Table 2. Result from linear models (LM) to test lipid contents depletion in different treatments over 21 days thermal stress exposure. **a** Comparison of lipid contents in each treatment before and after thermal stress. **b** Comparison of lipid depletion between treatments over 21 days thermal stress experiment.

a

**Treatments**	**estimate**	***SE***	***df***	***t*.ratio**	***p*.value**
**CC**	5.6	1.07	24	5.254	<.0001*
**CS**	4.15	1.07	24	3.889	0.0007*
**SC**	4.07	1.07	24	3.815	0.0008*
**SS**	2.72	1.07	24	2.551	0.017*

b

**Contrast**	**estimate**	***SE***	***df***	***t*.ratio**	***p*.value**
**CC-CS**	-0.728	0.754	24	-0.966	0.7699
**CC-SC**	2.842	0.754	24	3.77	0.0049*
**CC-SS**	2.168	0.754	24	2.876	0.0388*
**CS-SC**	3.57	0.754	24	4.736	0.0004*
**CS-SS**	2.896	0.754	24	3.842	0.0041*
**SC-SS**	-0.674	0.754	24	-0.894	0.808

Supplementary Table 3. Result from pairwise Wilcoxon test to test settlement rate between treatments. **a** Comparison of settlement rate of larvae in different treatments after 24h. **b** Comparison of settlement rate of larvae in different treatments after 48h.

a

Contrast	*p*.value
**CC-CS**	0.02*
**CC-SC**	0.3
**CC-SS**	0.08
**CS-SC**	0.05*
**CS-SS**	0.4
**SC-SS**	0.2

b

Contrast	*p*.value
**CC-CS**	0.06
**CC-SC**	0.2
**CC-SS**	0.8
**CS-SC**	0.8
**CS-SS**	0.06
**SC-SS**	0.2

Supplementary Table 4. Result from linear models (LM) to test settlement size between treatments.

Contrast	estimate	*SE*	*df*	*t*.ratio	*p*.value
**CC-CS**	0.1892	0.0298	76	6.339	<.0001*
**CC-SC**	0.105	0.0298	76	3.517	0.004*
**CC-SS**	0.1713	0.0298	76	5.74	<.0001*
**CS-SC**	-0.0843	0.0298	76	-2.823	0.0303*
**CS-SS**	-0.0179	0.0298	76	-0.6	0.9318
**SC-SS**	0.0664	0.0298	76	2.223	0.1262

Supplementary Table 5. Result from linear models (LM) to test growth of recruits in different treatments over two months.

Contrast	estimate	*SE*	*df*	*t*.ratio	*p*.value
**CC-CS**	-0.1868	0.0543	152	-3.444	0.0041*
**CC-SC**	0.0555	0.0543	152	1.023	0.736
**CC-SS**	-0.0132	0.0543	152	-0.243	0.9949
**CS-SC**	0.2424	0.0543	152	4.467	0.0001*
**CS-SS**	0.1736	0.0543	152	3.201	0.009*
**SC-SS**	-0.0687	0.0543	152	-1.267	0.5855

Supplementary Table 6. Kaplan-Meier log-rank test for comparison of survivorship of two moth old recruit from Stressed Fragment (SF) and Control Fragment (CF).

Contrast	*p*-value
**CC-CS**	1.20E-05*
**CC-SC**	0.666
**CC-SS**	4.80E-02*
**CS-SC**	4.40E-08*
**CS-SS**	0.012*
**SC-SS**	0.01*
